# Neuropsychological Rehabilitation in Mild and Moderate Alzheimer’s Disease Patients

**DOI:** 10.1155/2007/915816

**Published:** 2008-03-05

**Authors:** Renata Ávila, Isabel A. M. Carvalho, Cássio M.C. Bottino, Eliane C. Miotto

**Affiliations:** ^1^Old Age Research Group (PROTER)Department and Institute of PsychiatryFaculty of MedicineUniversity of Sao PauloSao PauloBrazil; ^2^Department of PsychiatryFaculty of MedicineUniversity of Sao PauloSao PauloBrazil; ^3^Division of Psychology and Department of NeurologyHospital das ClinicasUniversity of Sao PauloSao PauloBrazil

**Keywords:** Alzheimer’s disease, neuropsychological rehabilitation, memory, activities of daily living

## Abstract

*Objective:* The purpose of this study was to analyze the effect of a neuropsychological rehabilitation (NR) program on patients with Alzheimer’s disease (AD).

*Methods:* The sample was composed of 16 elderly outpatients who participated in an open trial with rivastigmine (6 to 12 mg/day) for 4 months and were randomized to 3 different groups: 1. group NR (*N* = 5), 2. individualized NR (*N* = 6) and 3. NR at home under supervision of a relative or caregiver (*N* = 5). All 3 groups fulfilled the same NR protocol consisting of a once a week session. Just before and after the 22 week period of rehabilitation, all patients were evaluated using psychiatric and functional scales, and neuropsychological tests by interviewers that did not participate in the cognitive training.

*Results:* The intervention did not produce any statistically significant change, but small gains were observed on some cognition tests, activities of daily living (ADL), and psychiatric symptoms in groups 1 and 2.

*Conclusion:* Group NR is recommended for reducing psychiatric symptoms, and individualized NR for improving ADL. NR at home either has no associated benefits, or the training sessions were not appropriately conducted by the caregiver. However, additional research with larger samples is necessary to confirm these observations.

## 1. Introduction

Substantial progress has been made in relation to neuropathology and diagnostic criteria of Alzheimer's disease (AD) over the last decades and also regarding molecular biology, geriatrics and pharmacological treatment. Nevertheless, thousands of patients and their relatives across the world still have to learn how to overcome the difficulties that arise with the progression of the disease.

Recent studies suggested that the combination of Neuropsychological Rehabilitation (NR) or Cognitive Rehabilitation (CR) alongside medication could be more effective in treating AD than medication alone, and maybe this might be the most effective way of treating these patients [2,19,20,29,30]. However, in spite of several accounts suggesting that rehabilitation is useful in treating patients with mild and moderate AD, there is insufficient scientific evidence in the literature from randomized controlled trials that have shown statistically significant results. Although some of these studies showed a slower decline or actual improvement on specific cognitive tests, the studies have not shown statistically significant benefits from the NR interventions [19,23,24,31,35].

The goal of this study is to report the effects of three different memory techniques and ADL training, in combination with medication in mildly to moderately impaired AD patients using three different interven-

ISSN 0953-4180/07/$17.00 © 2007 – IOS Press and the authors. All rights reserved tion groups, all of which receive the same training but in different contexts.

## 2. Methods

After the protocol was approved by the Ethics Committee and written informed consent was signed by each patient, eighteen elderly subjects ($\geqslant$ 60 years) diagnosed with mild to moderate AD according to National Institute of Neurological Communicative Disorders and Stroke (NINCDS/ADRDA) criteria [10] and ICD-10 [26] criteria and having used rivastigmine 6 to 12 mg/day, for more than 3 months, started an NR training program. All subjects were outpatients from the Old Age Research Group (PROTER), at the Department and Institute of Psychiatry, Faculty of Medicine, University of São Paulo, Brazil, previously submitted to a diagnostic work-up for dementia. Two patients could not continue the treatment, reducing the sample to sixteen patients.

The sample had 3 male (17.6%) and 13 female (82.4%) patients and the mean age was 73.8 years (SD: 4.8; range: 64–81). Seven patients were married (43.8%) and 9 widows (56.3%). Exclusion criteria were illiteracy; under 60 years of age; severe dementia with Clinical Dementia Rating (CDR) [4] $> 2$; history or clinical and/or radiological evidence of cerebrovascular disease; history or clinical evidence of other neurological disease; severe systemic or cerebral diseases; history of other psychiatric disease, including clinically significant, not controlled, depression; intolerance to rivastigmine; participation in another study with investigative drugs less than 4 weeks before this study started.

Before the initial assessment, subjects were ranked by age, education and severity of dementia and were pseudo-randomized in 3 groups also matched for age, schooling, and severity of dementia. They were followed for a 22 week-period: Group 1 – weekly group sessions of NR ($N = 5$); Group 2 – weekly individualized sessions of NR ($N = 6$); Group 3 – weekly sessions of NR at home monitored by a relative or a caregiver ($N = 5$). Pseudo-randomization was made by telephone by an assessor blind to the patient's group. The sociodemographic characteristics of the 3 groups of patients are presented in Table 1.

## 3. Assessment instruments

Just before and after the 22 nd week of NR period all patients were evaluated by the psychiatrist using the Bayer Scale for Activities of Daily Living (B-ADL) [14], Neuropsychiatric Inventory [14] (NPI), Hamilton Anxiety Scale (HAM-A) [16], Montgomery – Asberg Depression Rating Scale (MADRS) [33], MMSE and CDR (as well as the above, all patients carried out a Computed Tomography).

The following neuropsychological tests (Portuguese versions) were administered by the psychologist:

- Estimated Intelligence Quotient (IQ) based on Wechsler Abbreviated Scale for Intelligence (WASI) [5].
- Verbal memory: Recognition Memory Test for Words (RMW) [9], Logical Memory I and II – Wechsler Memory Scale – Revised (WMS-R) [6], Selective Remind Test (SRT) [12].
- Visual memory: Recognition Memory Test for Faces (RMF) [9], Visual Reproduction I and II (WMS–R) [6] .
- Alzheimer's Disease Assessment Scale – cognitive subscale (ADAS-COG) [32].
- Functional evaluation: Functional assessment [30], Memory Questionnaire of Daily Living (MQDL) for patients and relatives [23], Questionnaire of Quality of Life for patients and relatives (QOL) [22].

All the scales and tests were administered by interviewers that did not participate in the NR sessions and were blind to the patients' treatment group.

## 4. Rehabilitation methods

The NR group session was formed by 5 patients, and consisted in a 60-minute-session, once a week, coordinated by one psychologist and one speech-pathologist. The individual NR group was formed by 6 patients, and consisted of 40-minute-sessions, once a week, coordinated by the same psychologist and speech-pathologist. The 5 patients that formed the NR group at home were oriented by a relative or caregiver with an informative guide coming to the hospital only for the medical appointments and for the assessments. The relatives and caregivers of group 3 received an informative guide and were oriented on how to use it, before the beginning of NR. Relatives and caregivers also received a tele-

Table 1
Socio-demographic characteristics Pre-cognitive training of the three groups studied

| Variable | G1 = Group ($N = 5$) | G2 = Individual ($N = 6$) | G3 = Home ($N = 5$) |
|---|---|---|---|
| Gender | F = 4 (80 %)<br>M = 1 (20 %) | F = 5 (83.33 %)<br>M = 1 (16.67%) | F = 4 (80 %)<br>M = 1 (20%) |
| Age | 75.00 ± 4.42<br>(69–79) | 74.67 ± 3.88<br>(71–81) | 71.60 ± 6.23<br>(64–78) |
| Education (years) | 6.00 ± 3.08<br>(4–11) | 5.50 ± 3.27<br>(3–11) | 6.80 ± 3.27<br>(3–11) |
| Marital status | Married: 3 (60%)<br>Widowed: 2 (40%) | Married: 1 (16,66%)<br>Widowed: 5 (83,34%) | Married: 3 (60%)<br>Widowed: 2 (40%) |
| Rivastigmine | 6.60 ± 1.34<br>(6–9) | 7.75 ± 2.40<br>(6–12) | 8.10 ± 2.51<br>(6–12) |

F: female; M: male.

phone number to call if they had any queries or doubts about the training. The informative guide contained the same sequence of NR sessions performed by the other 2 groups of patients, and consisted of 40-minute-sessions, once a week.

During the NR program, the relatives and caregivers of all 3 groups participated in a 90-minute-group session, once a month, coordinated by the same professionals who worked with the patients.

## 5. Neuropsychological rehabilitation program

In the present study three different memory techniques were used, aiming at facilitating learning and recalling of material, motor movements, verbal association and categorization; ADL training with simulation of ordinary daily situations; and use of external aids like diaries, calendars and note books.

*Motor movements*: in order to learn the ordinary object's name from a list of words, all patients were trained to perform a motor movement or 'mime', as if they were using certain object, like making the gesture of combing one's hair to memorize 'comb'. When patients were asked to recall the object list, they were encouraged to repeat the mime and try to remember the object the action related to. After free recall, recognition tests were done. This technique was based on a prior study made by Moore et al. [34], where patients with AD should choose a particular motor movement that matched each member of the NR group's hobby, in order to improve the learning of the colleague's name. The authors used motor movement because it involves implicit memory, one kind of memory preserved in mild and moderate AD patients [15].

*Verbal association*: in order to improve learning and memory for a list of words, patients were trained to create a sentence or a short story with the words intended to be learned or remembered. Each sentence involved something particular and important for each one. Patients were encouraged to remember the sentence and the words. Just after, recognition tests were done. This technique aims to facilitate the new material codification through association with old and well learned information (for more detail about this subject see Deelman et al. 1994, cited by Miotto) [7].

*Categorization*: to improve learning and memory of a word list, patients were asked to organize the list, dividing it into categories (clothing, food, animal, etc). In order to recall the list with greater ease, they were asked to remember the categories first. As in the other training types, free recall and recognition tests were conducted. In this technique, the material to be memorized is organized into semantic categories. For this reason learning and recall is facilitated because categories work like clues do. This technique is cited by Clare and Wilson [17] and Glisky and Glisky [8].

*ADL training*: for this procedure three ADL were selected to be trained: telephone use, giving and receiving messages and diary use. For training these tasks, daily living situations were simulated, like making and receiving a real phone call using a telephone.

For all memory techniques training, different lists of six words were used in each session, in three different stages: in the first stage all words were read for the patients and just after they were asked to recall them. In the second stage, the words were read together with the presentation of the corresponding object, followed by recall. In the last stage, the memorization technique was taught, followed by recall. In each stage four repetitions were conducted. After these three stages, recognition test was done with 10 objects.

Each memory technique took three consecutive sessions. However, in each session the order of the three

Table 2
Results of the tests Pre and Post – NR

| Scales | Group 1 (Group) | | Group 2 (Individual) | | Group 3 (Home) | |
|---|---|---|---|---|---|---|
| | pre | post | pre | post | pre | post |
| MMSE | 20.00 (4.00) | 20.00 (5.61) | 20.83 (5.04) | 21.67 (5.54) | 18.40 (5.08) | 15.20 (5.61) |
| CDR | CDR 1:4 CDR 2:1 | CDR 1:3 CDR 2:2 | CDR 1:5 CDR 2:1 | CDR 1:5 CDR 2:1 | CDR 1:4 CDR 2:1 | CDR 1:2 CDR 2:2 CDR 3:1 |
| ADAS-COG | 25.40 (5.64) | 22.40 (6.35) | 23.67 (12.56) | 22.83 (13.36) | 32.40 (14.54) | 37.40 (21.87) |
| HAM-A | 4.00 (2.45) | 3.40 (1.34) | 5.50 (5.24) | 6.83 (5.60) | 5.60 (2.41) | 5.60 (2.30) |
| MADRS | 5.60 (2.88) | 4.80 (2.17) | 7.33 (3.50) | 8.33 (4.27) | 7.20 (4.15) | 12.40 (7.40) |
| NPI | 5.60 (2.30) | 12.60 (5.68) | 9.50 (5.92) | 14.16 (19.50) | 12.00 (9.19) | 19.00 (14.57) |
| B-AVL | 5.89 (1.56) | 6.22 (1.88) | 5.49 (1.68) | 4.99 (1.82) | 5.90 (1.55) | 6.69 (3.36) |

stages described above was modified. At the end of the program, the techniques were reviewed. As well as the above, ADL training sessions were conducted. Each task was trained during three consecutive sessions, inserted between the memory training, and then reviewed at the end of the treatment. In the last session, there was one socialization activity with patients and relatives of the three groups, all participating in a party celebrating the end of the NR program.

*Group support intervention for relatives and caregivers*: this monthly group session focused on orientation about AD course and prognosis, counseling and support. All participants were encouraged to share their experiences and coping strategies.

## 6. Procedures

Before the beginning of NR program all selected patients were evaluated by a standard protocol. The assessment was done with the patient and a family member during a 40 minute session, and included the psychiatric protocol. The evaluation was carried out at baseline, in order to evaluate the treatment effect, comparing their scores with a post treatment assessment. After this assessment, a psychologist evaluated all patients during two sessions of 1 hour each. All patients were accompanied by a family member which is essential in order to answer the QOL and MQDL scales. However, they remained in a room separated from the patients.

### *6.1. Statistical analysis*

A 5% (0.05) significance level was adopted. The Statistical Package for Social Science (SPSS) [21] was selected in order to carry out the analyses.

Initially, descriptive analyses were performed including mean, standard deviation, range and frequencies on the data. Analyses of Variance (ANOVA) and Tukey Test were applied in order to find any significant differences between the three groups in terms of age, education, MMSE, HAM-A, MADRS, NPI, B-AVD, ADAS-COG and Estimated IQ. All variables showed a normal distribution.

Student's t-test were carried out with the continuous variables. For the categorical variables, the Wilcoxon analysis was selected.

In order to compare the differences between the three groups, a series of ANOVAs were performed using Tukey as a "Post-Hoc" method.

Besides canonical statistical procedures, the effect size (ES) of scales and memory tests for each group were calculated, according to the formula: ES = mean post-treatment – mean/pre-treatment. Rockwood, Joyce and Stolee [16] stated that the ES takes into account the within-group variance on performance or behavior at baseline and that a larger number represents a larger therapeutic effect.

## 7. Results

At baseline there were no statistically significant differences between the three groups, but on MMSE,

Table 3
Results of the neuropshychological tests Pre and Post – NR

| Tests | Group 1 (Group) | | Group 2 (Individual) | | Group 3 (Home) | |
|---|---|---|---|---|---|---|
| | pre | post | pre | post | pre | post |
| Logic M. I | 5.80 | 6.80 | 6.83 | 6.50 | 2.60 | 3.40 |
| | (3.03) | (2.49) | (9.09) | (9.07) | (2.70) | (3.44) |
| Logic M. II | 0.00 | 0.40 | 3.67 | 3.50 | 0.00 | 0.20 |
| | (0.00) | (0.89) | (8.98) | (8.08) | (0.00 | (0.45) |
| Visual R. I | 14.80 | 13.00 | 13.83 | 3.60 | 8.20 | 11.60 |
| | (7.46) | (5.92) | (3.60) | (7.23) | (7.46) | (9.24) |
| Visual R. II | 0.00 | 0.00 | 1.33 | 2.67 | 0.00 | 0.40 |
| | (0.00) | (0.00) | (2.80) | (6.53) | (0.00) | (0.89) |
| SRT | 26.00 | 24.60 | 25.33 | 26.00 | 21.00 | 23.20 |
| | (9.41) | (4.93) | (16.57) | (18.01) | (10.89) | (17.43) |
| RMF | 27.60 | 28.60 | 28.00 | 29.33 | 25.80 | 24.40 |
| | (7.27) | (5.13) | (5.90) | (7.17) | (2.47) | (3.78) |
| RMW | 26.60 | 31.00 | 32.67 | 31.67 | 29.20 | 25.20 |
| | (3.71) | (6.04) | (8.91) | (8.19) | (4.32) | (14.81) |
| Functional E. | 5.40 | 4.40 | 5.00 | 6.00 | 3.20 | 2.60 |
| | (0.55) | (0.55) | (1.55) | (2.19) | (3.03) | (3.21) |
| MQDL – Patient | 99.20 | 101.20 | 132.67 | 105.00 | 134.00 | 99.80 |
| | (18.70) | (40.71) | (35.07) | (26.18) | (43.32) | (33.58) |
| MQDL – Relatives | 133.60 | 159.60 | 172.50 | 170.67 | 184.20 | 188.60 |
| | (19.46) | (28.06) | (46.97) | (51.00) | (4.78) | (86.40) |
| QOL – Patient | 36.80 | 37.40 | 35.17 | 34.00 | 34.80 | 34.60 |
| | (2.86) | (2.51) | (2.23) | (2.28) | (10.71) | (10.92) |
| QOL – Relatives | 30.00 | 31.20 | 29.33 | 29.83 | 31.40 | 30.60 |
| | (4.42) | (5.02) | (5.61) | (6.08) | (7.57) | (4.45) |

Logic M I and II: Logic Memory I and II; Visual R.I and II: Visual Reproduction I and II; SRT: Selective Reminding Test; RMF: Recognition Memory Face; RMW: Recognition Memory Words, Functional E.: Functional Evaluation; MQDL: Memory Questionnaire of Daily Living; QOL: Questionnaire of Quality of Life.

ADAS-COG, NPI and IQ, group 3 subjects had the lower result.

The quantitative analyses regarding the NR program are presented in Tables 2 and 3, where scales and tests scores applied to patients and their families, pre and post treatment are shown. The intervention did not produce any statistically significant change in any group. But some observations could be made and will be discussed in the next section. The results of ES on the scales, memory and ADL tests on the 3 groups studied are shown in Table 4, and no significant effects were observed.

## 8. Discussion

In the present study a NR program was applied in three different intervention groups. The intervention did not produce any statistically significant change; however, a small improvement was observed in cognition, functional aspects, and psychiatric symptoms depending on the group, allowing some observations.

The lack of significant results may have been due to the small number of patients, while studies of cholinesterase inhibitors in AD have showed significant effect of treatment when very large number of patients have been studied.

On the scales that evaluate global functioning, MMSE and ADAS-COG, it was observed that the patients who underwent NR in group or individually had stabilized or slightly improved their scores, while those who conducted training at home, had a decline in both scales. From the 5 patients of group 3, relatives and caregivers of 3 (60%) patients reported having conducted all week sessions as described in the guide, and 2 (40%) reported that they had not done this in 50% of the training sessions.

The effects of the specific memory training techniques were not seen in any of the three groups regarding the neuropsychological reassessment performed. This finding may suggest that the memory training brings no positive effects, or cognitive tests used here are not sufficiently sensitive to capture these positive effects, or there were no sufficient numbers of patients in any group to show a positive effect.

In addition, standardise neuropsychological tests are usually not sensitive to changes after cognitive or NR.

Table 4
ES of the scale and tests of the 3 groups studied

| Scales and tests | Group 1 (Group) | Group 2 (Individual) | Group 3 (Home) |
|---|---|---|---|
| MMSE | 0 | 0.1666 | −0.5517 |
| ADAS-COG* | −0.5319 | − 0.0668 | 0.343 |
| HAM-A* | −2.448 | 0.2538 | 0 |
| MADRS* | −0.2777 | 0.2857 | 1.2530 |
| NPI* | 3.0434 | 0.7871 | 0.7616 |
| BAYER-ADL* | 0.2756 | −0.2976 | 0.5096 |
| Function evaluation | −1.8181 | 0.6451 | −1.1980 |
| SRT | −0.1487 | 0.0404 | 0.2020 |
| Logic memory I | 0.330 | −0.363 | 0.363 |
| Logic memory II | 0 | −0.018 | 0 |
| Visual reproduction I | −0.241 | 1.575 | 0.455 |
| Visual reproduction II | 0 | 0.478 | 0 |
| RMF | 0.137 | 0.225 | −1.40 |
| RMW | 1.185 | −0.112 | −0.925 |

SRT: Selective Reminding Test; RMF: Recognition Memory Face; RMW: Recognition Memory Words.
*On this scales negative results indicated an improvement.

One possible explanation is that they measure impairment instead of disability or handicap. The last two levels should be the focus of NR outcome. This may mean that some effects, of possible relevance to daily life, are missed. Davis et al. [31] noted an improvement, during AD patients training, in recall of personal information, face-name recall, and performance on the Verbal series Attention Test. However, the improvement did not generalize to additional neuropsychological measures and was not captured by the outcome measured in their study. The same was observed in this study.

ADL training conducted in group sessions or at home, showed a reduced learning capacity as demonstrated by lack of improvement in ADL. However, the patients who underwent individual NR demonstrated small improvements (Functional evaluation: pre 5 and post 6, out of 9 points; B-ADL: pre 5.49 and post 4.99, out of 10 points) possibly because in individual training sessions, the specific difficulties of each one are dealt with directly. As improvement was not noticed in the patients who underwent home training, we can assume that the training sessions were either not conducted properly or there were non-random differences between groups, due to the small numbers of patients in each group.

As for psychiatric symptoms, such as anxiety and depression, group 1 (Group NR) only showed a positive effect (HAM: pre 4.00 and post 3.40; MADRS: 5.60 post 4.80), while the other intervention group did not. We can infer that being with other people who have similar difficulties, as well as being part of a group, helps to reduce the symptoms of anxiety and depression.

The pre and post results of MQDL and QOL instruments were not revealing, which indicates that the QOL of these patients and relatives does not improve with this type of intervention, and that the memory complaints can improve in some aspects, but deteriorate in others.

A comparison can be made between the current results and those from a previous study performed by Avila et al. [30] in a similar sample of patients undergoing weekly group and individualized sessions, as well as informative group sessions for their families. In this previous study, significant improvement was verified in ADL ($p = 0.04$), and a small improvement in memory and psychiatric symptoms. It is likely that in group sessions the focus is on enhancing social, psychological and behavioural aspects, whereas in the individual sessions more attention is given to the ADL and memory training.

During the NR sessions with the patients, and in the support groups for relatives and caregivers, some observations were made. It was noted that in some sessions of the group therapy, specifically during memory training, a lack of enthusiasm and even a certain resistance to learning some of the techniques, was noticed. This lack of enthusiasm was proportional to the ability to respond to the memory training. Those who were able to benefit from the repetitions as well as the techniques were more engaged with the treatment, while those who had more difficulties were less willing to comply.

One family member and one caregiver who conducted the training with the patient at home, informed us that in order to do these training sessions, they had to choose a good moment, as there were times when the patients did not want to do the training. In this way, maybe these techniques should be limited to individual sessions with patients who had mild cognitive deficits. Perhaps this lack of engagement occurs due to the difficulty in seeing the practical side of this training, even though this was explained and exemplified in each session. In contrast, patients who received individualized sessions showed greater enthusiasm.

After considering each technique individually, it was verified that the one that used motor movements only had positive results for those patients who actually carried out the mime in a repetitive way. The more reserved and unenthusiastic patients did not benefit from this type of technique. This was observed both in the group and in the individualized training sessions. The categorization technique showed good results in both the group and individualized training sessions. The patients had greater facility in learning and benefited more from this strategy then other strategies. It was interesting to note that the technique that proved to be the most difficult was the verbal association, either in generating the associations or during the delayed recall of words and phrases. This difficulty was observed both in the group and the individual sessions.

During ADL training sessions, the patients were more participating and interested. It was also noted that these were better carried out in individual sessions, where greater attention could be given to the specific difficulties of each person. However, in group sessions, it was not uncommon to see a patient helping another one with the carrying out a task. This was a positive factor in augmenting sociability. In relation to group 3, the family members and caregivers did not comment in detail on these training sessions.

A comparison with similar studies could not be done due to the non-existence in the literature of any study with a similar objective of verifying the difference in the effect of NR in these three specific formats of intervention. However, when looking at the broader aim of the study, which was to verify the effect of memory training techniques and of ADL in patients with mild to moderate AD, some comparisons can be made.

First, a small number of published controlled trials indicate similar results, showing some improvement on cognitive [19,18] and ADL tasks [27,28,30], and reduced psychiatric symptoms [2,34].

Further comparisons could be done with studies split into the types of intervention carried out (group, individual or at home). The hypothesis stipulated in this study, that group NR improves or maintains the global functioning and psychiatric symptoms of individuals in the group, corroborates earlier studies [2,34]. Evidence of improvement in ADL in individual training also has been seen in studies such as the only reported by Zanetti et al. [27,28]. Cognitive stimulation training conducted at home by the spouse of the patient with AD has been studied by Quayhagen et al. [23,24] showing significant improvement after intervention, and the tendency of improvement when compared to the group studied with a control and placebo.

## 9. Conclusion

The results of the present study are in line with other randomized controlled studies already conducted with AD patients where statistical analyses showed that improvements were not significant. This makes definite conclusions difficult to be drawn from them. On the other hand, the lack of statistical significance does not exclude the possibility of reaching some other observations from the present study that suggest that (1) group or individual memory training are more likely to result in stabilization or even in small improvement of the global efficiency of the patients, than home programs; (2) NR individually seems to be more effective for training ADL, while (3) group NR seems to be better to reduce patient's anxiety and depression.

The usefulness of memory training techniques where patients cannot generalize this to other aspects of their life continues to be a relevant issue. An alternative could be to restrict the training to daily problem solving with the help of external aids and improvement in ADL performance. Another alternative could be to evaluate the efficiency of other memory techniques. In addition, one should question the validity of training programs at home with families or caregivers, where there is no proper monitoring by a professional, as some of these patients tend not to follow the treatment completely.

In conclusion, even if the quantitative data have not been encouraging, one should bear in mind that the population studied suffers from a degenerative disease. The cognitive function stabilization and small improvements or stabilization in ADL performance or in psychiatric symptoms are not dispensable gains. The implication that further non-pharmacological studies with larger samples are necessary is a result in itself, in order to promote a better understanding of the strengths and weaknesses of the various types of intervention in non-pharmacological treatment.

## Acknowledgements

We thank our patients, their relatives and caregivers who participated in this study. This study was supported by a grant from the Fundação de Amparo a Pesquisa do Estado de Sao Paulo (FAPESP) grant nr. 2001/13834-0.

## Appendix

Functional Test

Patient's name: Date of evaluation:

Chores:

1. Receive and take note of a message by phone. Tell the patient: "when the phone rings you should answer it" – "Mr.(s) has a doctor appointment at the hospital next Monday at three o'clock"

a) answers the phone spontaneously
b) says that he/she will take the message spontaneously
c) takes the message before hanging up the phone
d) checks that he/she wrote the message down correctly before hanging up the phone

Observations:

2. Take note of an appointment in the diary. Tell the patient: "I will tell you an appointment and you will take note of this appointment in your diary: "Mr.(s) has a dentist appointment next Wednesday at two o'clock"

a) manages to locate today's date in the diary without any help
b) takes note of the appointment on the right day, that is, the day of the appointment
c) takes note of the complete details of the appointment

Observations:

3. Write a note giving someone a message. Tell the patient: "I will give you a message for you to tell the other person: "Son, the cleaning lady called informing that she will not be coming to work tomorrow"

a) takes note of the message alone
b) takes note of the message completely

Observations:
